# Association between constipation and risk of coronary heart disease: a systematic review and meta-analysis of cohort studies

**DOI:** 10.3389/fcvm.2025.1622801

**Published:** 2025-12-04

**Authors:** Feng Tang, Tianjun Zhao, Peiwen Dong, Kaidi Sun, Xiaobin Sun, Qiong Wang

**Affiliations:** 1 Department of Gastroenterology, The Affiliated Hospital of Southwest Jiaotong University, The Third People’s Hospital of Chengdu, Chengdu, China; 2 Department of Cardiology, The Affiliated Hospital of Southwest Jiaotong University, The Third People’s Hospital of Chengdu, Cardiovascular Disease Research Institute of Chengdu, Chengdu, China

**Keywords:** constipation, coronary heart disease, myocardial infarction, cohort studies, meta-analysis

## Abstract

**Objective:**

This systematic review and meta-analysis aimed to evaluate the association between constipation and risk of coronary heart disease (CHD).

**Methods:**

We systematically searched PubMed, Web of Science, and Cochrane Library until 28 February 2025. Published cohort studies reporting quantitative association measures for CHD in constipated vs. non-constipated individuals were included. The heterogeneity was assessed via the chi-square test based on Cochrane Q statistics. *I*^2^ > 50% or *Q*-test *p* < 0.05 indicated substantial heterogeneity, warranting random-effects modeling; otherwise, fixed-effects models were implemented. Subgroup evaluations were conducted for study design type, region, category of CHD, follow-up duration, and gender.

**Results:**

Nine studies involving 283,070 constipation cases and 3,343,120 controls were analyzed. Constipation was associated with a 10% increased CHD risk [hazard ratio (HR] = ).10, 95% confidence interval (CI): 1.05–1.15]. Statistical heterogeneity (*I*^2^ = 42.5%, *p* = 0.03) was observed in the present study. Subgroup analyses revealed a stronger association with myocardial infarction (HR = 1.14, 95% CI: 1.05–1.23). Notably, constipation showed no CHD risk elevation in women (HR = 1.04, 95% CI: 0.98–1.11), with reduced residual heterogeneity (*I*^2^ = 30.2%, *p* = 0.177).

**Conclusion:**

Our meta-analysis identified a significant positive association between constipation and CHD risk, particularly myocardial infarction. These findings suggest that constipation may either accelerate the pathological processes underlying CHD or that both conditions share common etiological pathways, warranting further mechanistic and interventional studies.

## Introduction

Cardiovascular diseases (CVDs), particularly coronary heart disease (CHD), persist as the foremost contributor to global mortality. CHD continues to pose a critical public health challenge, with its burden escalating worldwide. Epidemiological surveillance reveals a sustained upward trajectory in CHD-related mortality since 1990, culminating in 9.14 million deaths and 197 million prevalent cases in 2019 (1). The Global Burden of Disease Study quantifies disease burden attributable to 88 modifiable risk factors. The results showed that key cardiovascular risk drivers include high blood pressure, dietary risks, high LDL cholesterol, air pollution, tobacco, high body mass index, high fasting plasma glucose, and kidney dysfunction among others (2). Emerging epidemiological evidence suggests that constipation may represent a novel modifiable risk factor for cardiovascular disease (3–6).

Constipation, with a global prevalence exceeding 10%, emerges as both a common clinical manifestation of gastrointestinal dysmotility and a significant public health priority worldwide (7). This condition manifests through suboptimal defecation experience, resulting from either reduced bowel movement frequency, straining during evacuation, incomplete rectal emptying, or the co-occurrence of colonic hypomotility and pelvic floor dyssynergia (8). A growing body of evidence has recently emerged examining the potential association between constipation and the risk of CHD. Although Ma et al. reported that increased frequency of bowel movements was positively associated with higher CHD risk, other studies have indicated a significant relationship between constipation and the development of CHD (3, 4, 9). Despite the increasing number of epidemiological studies on this topic, the nature and direction of the association between constipation and CHD risk remain inconsistent and subject to ongoing debate. This systematic review and meta-analysis were therefore conducted to quantitatively synthesize existing evidence regarding their association and evaluate the association between constipation and risk of CHD.

## Methods

### Search strategy

This systematic review and meta-analysis were executed in full compliance with Preferred Reporting Items for Systematic Reviews and Meta-Analyses reporting standards. The study protocol was prospectively registered with the International Prospective Register of Systematic Reviews (registration ID: CRD42024615729). A comprehensive literature search spanning PubMed, Web of Science, and the Cochrane Library was conducted through 28 February 2025 using the following Boolean search syntax: (“cardiovascular disease” OR “cardiovascular diseases” OR “CVD” OR “cardiovascular events” OR “heart disease” OR “heart diseases” OR “coronary artery disease” OR “coronary heart disease” OR “ischemic heart disease” OR “CHD” OR “myocardial infarction” OR “angina pectoris”) AND (“constipation” OR “bowel movement frequency”).

### Inclusion criteria

Studies were eligible for inclusion if they met the following criteria: (1) peer-reviewed original research articles published in full text; (2) conducted in human populations; (3) studies defining constipation exposure through validated diagnostic criteria; (4) comparative design with constipated and non-constipated cohorts, reporting quantitative association measures [hazard ratio (HR), relative risk (RR), or odds ratio (OR)] with 95% confidence intervals (CIs), or providing sufficient data for their calculation.

### Exclusion criteria

The following study types were systematically excluded during screening: (1) animal studies; (2) non-longitudinal study designs (cross-sectional analyses, case reports), gray literature (conference abstracts), and non-peer-reviewed materials (reviews, editorials); (3) non-English publications; (4) studies lacking extractable outcome metrics (risk ratios with 95% CIs or raw data for their computation).

### Data abstraction and quality assessment

Dual-independent data extraction was performed by two investigators using a predefined extraction template, with discrepancies adjudicated through iterative discussion. In cases where multiple studies might have utilized the same registry or database, only one study per distinct cohort was retained to avoid double-counting. Extracted parameters encompassed: first author's name, publication year, country, study design, participants, definition of constipation, sample sizes in comparison groups, follow-up duration, and adjusted confounders. Methodological rigor was evaluated via the Newcastle-Ottawa Scale (NOS), employing its tripartite assessment framework: (1) cohort selection (0–4 points), (2) intergroup comparability (0–2 points), and (3) exposure ascertainment (0–3 points). Studies were stratified by total NOS scores (maximum 9 points), with higher scores denoting superior methodological quality.

### Statistical analysis

For each study, we pooled effect estimates as the HR and their 95% confidence intervals (95% CIs). Logarithmic transformation was applied to HR values to calculate standard errors. Heterogeneity among studies was quantified via Cochran's *Q* statistic (chi-square test) and *I*^2^ index (percentage of total variation attributable to heterogeneity). *A priori* thresholds for heterogeneity interpretation were established: *I*^2^ > 50% or *Q*-test *p* < 0.05 indicated substantial heterogeneity, warranting random-effects modeling; otherwise, fixed-effects models were implemented. Galbraith radial plots identified outlier studies contributing to residual heterogeneity. To explore possible explanations for homogeneity and test the robustness of the association between constipation and risk of CHD, we preplanned subgroup analyses by study design type, region, category of CHD, follow-up duration, and gender. Meanwhile, we conducted sensitivity analyses based on leave-one-out iterative recalculation of pooled estimates. Funnel plot asymmetry was evaluated through Begg's rank correlation and Egger's weighted regression tests. A trim-and-fill non-parametric analysis of publication bias were used to address potential publication bias. All analyses were conducted in Stata 18.0 (Stata Corp, College Station, TX, USA) with two-tailed *p* < 0.05 defining statistical significance.

## Results

### Study characteristics

**Figure 1:**
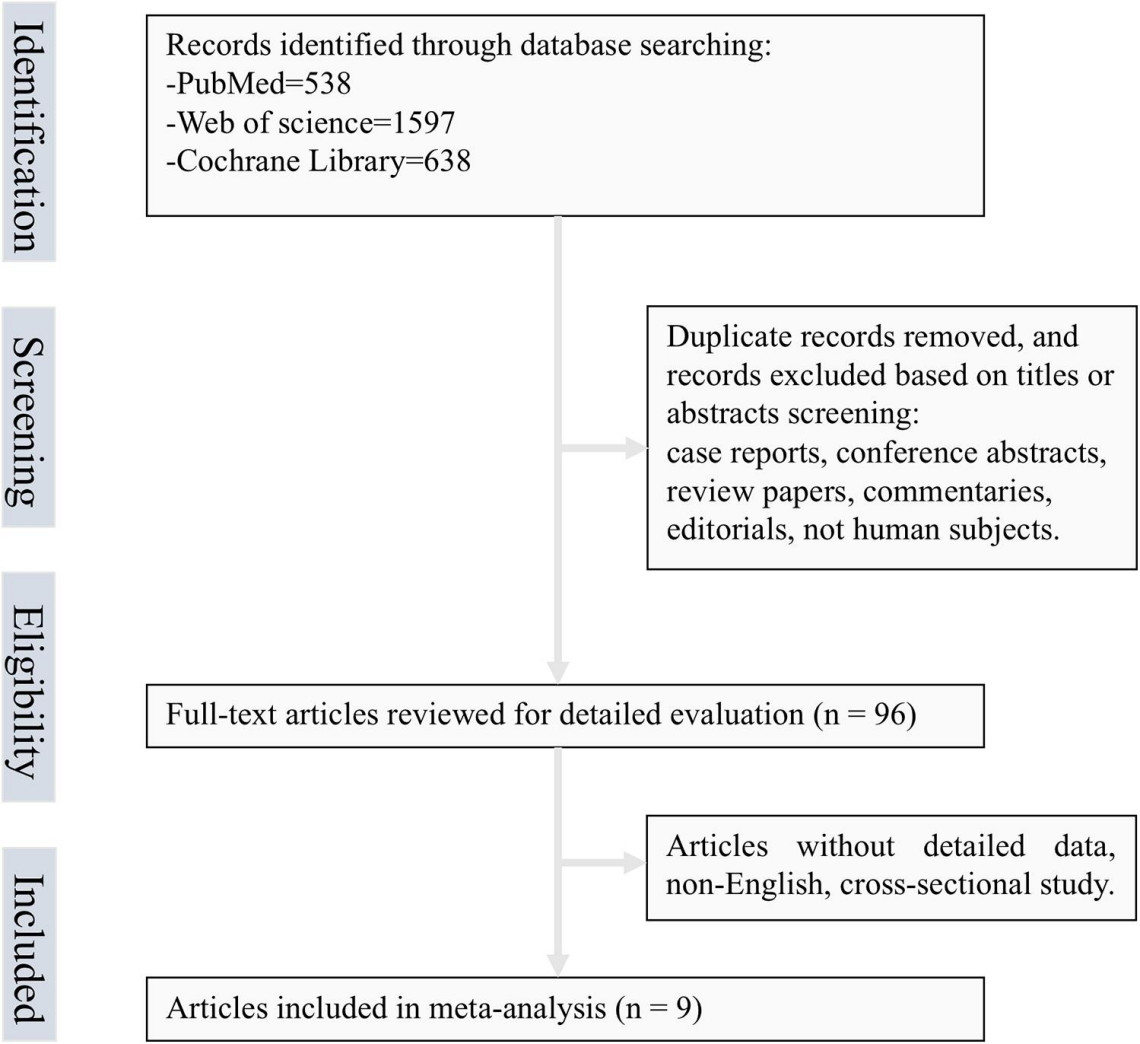
Flow chart of selected articles.

The systematic review initially retrieved 2,773 records from electronic databases. After title/abstract screening and eligibility assessment (Figure 1), nine studies comprising 283,070 constipation-affected participants and 3,343,120 non-constipated controls were ultimately included. During screening, we excluded three studies that provided only OR on constipation and CHD risk, which we thought would affect our inference of causality (10–12). Heterogeneous diagnostic criteria for constipation across studies were systematically documented. Table 1 details the cohort characteristics of included investigations. The meta-analysis incorporated eight cohort studies and one nested case-control study, the latter included due to its unique exposure–outcome ascertainment framework (3–6, 9, 13–16). Geographically, the studies comprised four US-based investigations, four from Asian nations, and one European cohort. Sex distribution analysis revealed seven studies with mixed-sex cohorts and two exclusively female populations. Methodological rigor was evaluated using the NOS, with all nine studies meeting the predefined quality threshold (NOS score ≥ 6). Comprehensive risk-of-bias assessments are shown in Table 2. In addition, Supplementary Table S1 describes a concise summary of the bias risk evaluations for all included studies.

**Table 1:** Characteristics of included studies in the meta-analysis.

Study (first author)	Year	Country	Study design	Participants	Definition of constipation	Duration of follow-up (years)	Number with constipation	Number without constipation	Adjustment for confounding variables
Salmoirago-Blotcher et al.	2011	USA	Prospective cohort study	Postmenopausal women	Defined as “difficulty having bowel movements” over the previous 4 weeks, was rated using a scale ranging from none (did not occur), mild did not interfere with usual activities), moderate (interfered somewhat with usual activities), or severe (symptom was so bothersome that usual activities could not be performed)	Median follow-up: 6.9 years	Mild: 18,790 Moderate: 5,391 Severe: 1,167	47,699	Adjustment for demographics, risk factors, dietary factors, medications, frailty and other psychological variables
Choung et al.	2016	USA	Prospective, population-based nested case-control study	Community residents	Rome III criteria	Four-year period	307	2020	Adjusted for age and gender
Honkura et al.	2016	Japan	Prospective population-based study	The subjects were all National Health Insurance beneficiaries, aged 40–79 years	Defined as the defecation frequency groups: ≤1 time/4 days	13.3 years of follow-up	835	36,158	Adjusted for age, sex, body mass index, hypertension, diabetes mellitus, smoking status at baseline, alcohol consumption, education level, time spent walking per day, baseline job status, stress awareness, marital status, fruit and vegetable intake
Kubota et al.	2016	Japan	Prospective cohort study	Subjects aged 40–79 years, without a history of CVD or cancer	Bowel movement once every 4 or more days	19 years of follow-up	Men: 316 Women: 1,861	Men: 26,346 Women: 28,738	Adjusted for age, history of hypertension, history of diabetes, body mass index, alcohol intake, smoking status, depressive symptoms, perceived mental stress, walking, sports, energy-adjusted dietary fiber intake, living in urban areas and menopausal status for women
Ma et al.	2016	USA	Prospective cohort study	Women free from CVD and cancer	Frequency of bowel movements: every 3–4 days or every 5 days or less	Up to 30 years of follow-up	Every 3–4 days: 6,348 Every 5 days or less: 1,067	54,264	Adjusted for age, ethnicity, menopausal status, smoking status, physical activity, family history of myocardial infarction, baseline history of hypertension, hypercholesterolemia, ulcerative colitis, cholecystectomy, use of multivitamin, aspirin, other nonsteroidal anti-inflammatory drugs, thiazide diuretics, thyroid hormone, alcohol intake, Alternate Healthy Eating Index score, dietary intake of total fiber, total energy intake, body mass index, and baseline history of diabetes
Sumida et al.	2019	USA	Retrospective cohort study	Veterans with an estimated glomerular filtration rate ≥ 60 mL/min/1.73 m^2^	Defined as either having ≥2 prescriptions of laxatives of ≥30-day supply each, that were 60–365 days apart during the baseline period based on information obtained from VA Pharmacy dispensation records; or having at least two diagnoses for constipation, as identified by the ICD-9-CM, that were ≥60 days apart	Median follow-up of 6.7 years	237,855	3,121,798	Multivariable adjustments for demographics, prevalent comorbidities, medications, and socioeconomic status
Sundbøll et al.	2020	Denmark	Population-based matched cohort study	Constipated patients in contact with the healthcare system-excluded patients with a previous or concurrent inpatient or outpatient diagnosis of any of the study outcomes	Diagnosed according to the International Classification of Diseases, Eighth Revision (ICD-8) through 1993 and 10th Revision (ICD-10) thereafter	10 years of follow-up	83,239	832,384	Controlled for matching factors (age, sex, and calendar year) and adjusted for hypothyroidism, hyperthyroidism, pregnancy within 90 days before the index date, depression, Parkinson's disease, multiple sclerosis, colon, rectal and anal cancer, other gastrointestinal cancers, Crohn's disease, ulcerative colitis, paralytic ileus, chronic pulmonary disease, valvular heart disease, diabetes mellitus, hypertension, hypercholesterolemia, obesity, chronic kidney disease, liver disease, alcoholism-related disorders, medications associated with constipation, and cardiovascular drugs
Yang et al.	2020	China	Population-based prospective cohort study	Without cancer, heart disease or stroke at baseline	Frequency of bowel movements: less than three times a week	A median of 10 years	21,148	373,054	Adjusted for sex; level of education; occupation; household income; marital status; family history of certain diseases; smoking status; total physical activity level; alcohol consumption; intake frequency of fresh vegetables, fresh fruit and red meat; BMI; waist circumference; prevalent hypertension, and diabetes at baseline
Park et al.	2025	Republic of Korea	Retrospective cohort study	Patients undergoing maintenance hemodialysis	Using the total number of prescribed laxatives ≥180 during the 1-year baseline period	A median follow-up of 5.4 years	9,133	26,097	Adjusted for age, sex, dialysis vintage, comorbidities (diabetes mellitus, hypertension, ischemic heart disease, congestive heart failure, cerebrovascular disease, atrial fibrillation or flutter, and malignancy)

**Table 2:** Results of the quality of studies in meta-analysis using the NOS.

Study	Selection				Comparability	Exposure		
	Adequate definition of cases	Representativeness of the cases	Selection of controls	Definition of controls	Control for important factor^a^	Ascertainment of exposure	Same method of ascertainment for cases and controls	Non-response rate
Salmoirago-Blotcher et al.	★	☆	★	★	★★	★	★	★
Choung et al.	★	★	★	★	★☆	★	★	★
Honkura et al.	★	★	★	★	★★	★	★	★
Kubota et al.	★	☆	★	☆	★☆	★	★	★
Ma et al.	★	☆	☆	★	★★	★	★	★
Sumida et al.	★	☆	★	★	★☆	★	★	★
Sundbøll et al.	★	☆	★	★	★★	★	★	★
Yang et al.	★	☆	★	★	★☆	★	★	★
Park et al.	★	☆	★	★	★☆	★	★	★

a A maximum of two stars can be allotted in this category, one for age, the other for other controlled factors.

### Constipation and risk of CHD

**Figure 2:**
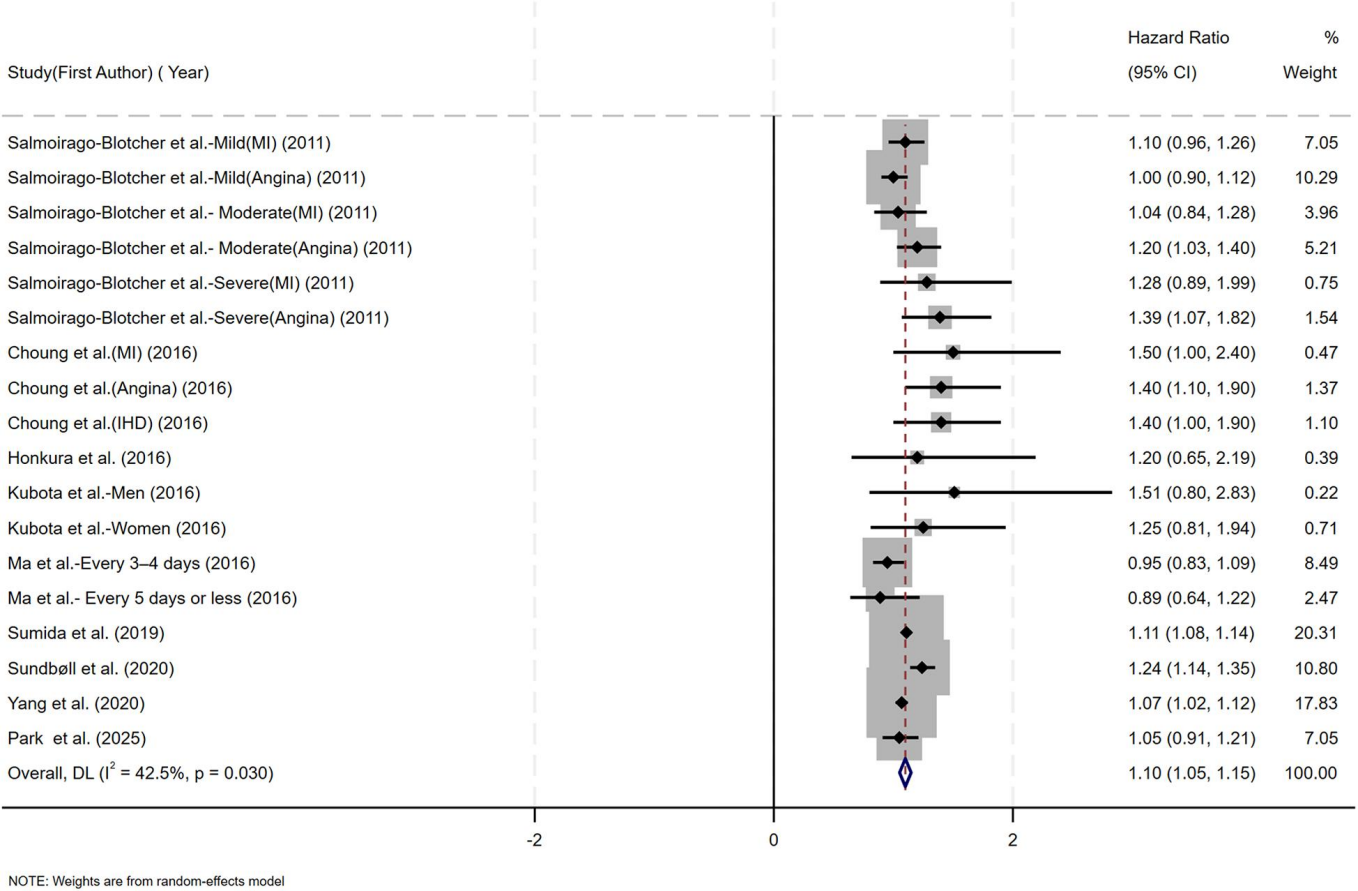
Forest plot of the meta-analysis of included studies assessing the association between constipation and CHD risk.

**Figure 3:**
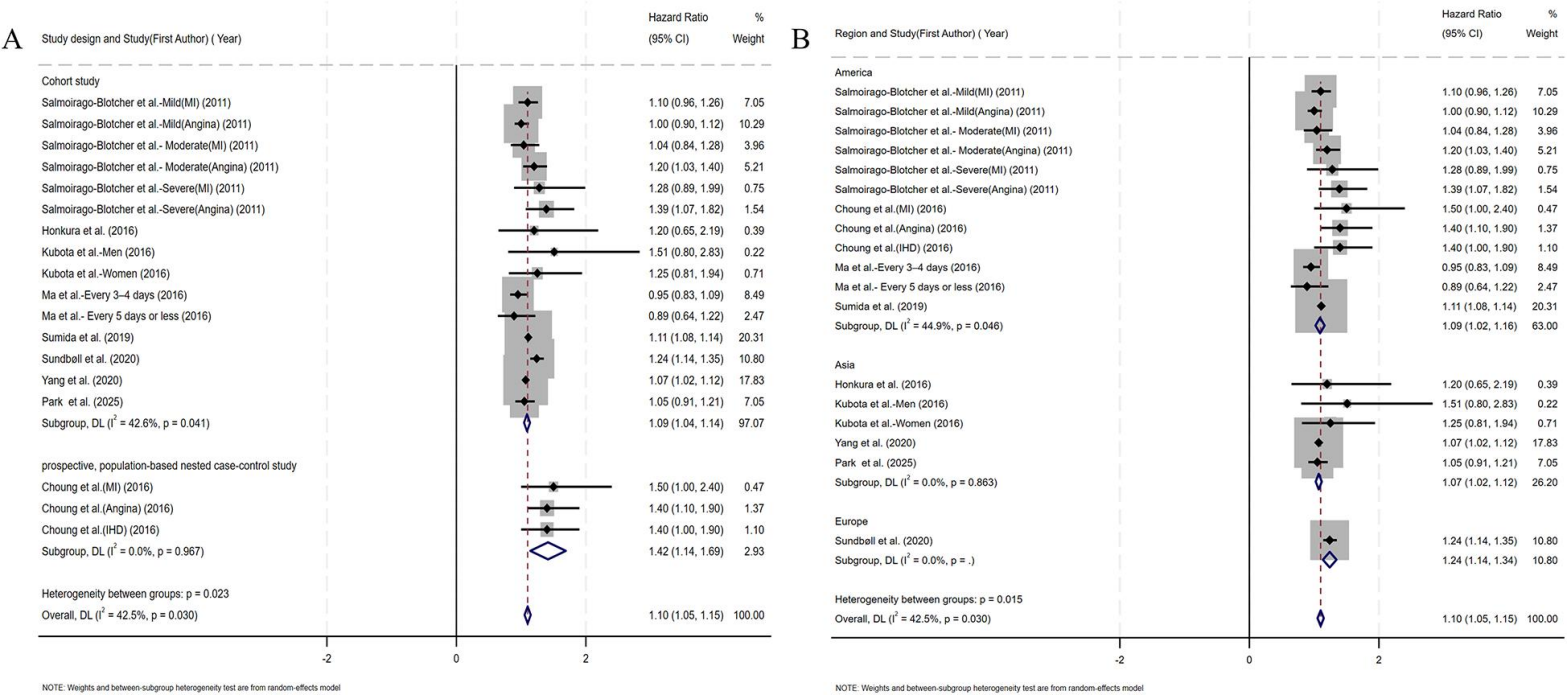
**(A)** Forest plots of subgroup studies regarding the association between constipation and CHD risk based on different study design; **(B)** forest plots of subgroup studies regarding the association between constipation and CHD risk in different regions.

Figure 2 presents the multivariable-adjusted hazard ratios (HRs) for CHD risk associated with constipation across included studies. Pooled analysis demonstrated a statistically significant 10% increased CHD risk among constipated participants vs. controls (HR=1.10, 95% CI: 1.05–1.15). Three studies reporting solely ORs were excluded from primary analysis due to the inherent limitations of ORs in causal inference. However, a meta-analysis incorporating these OR-based and HR-based studies (total *N* = 12) demonstrated strengthened pooled effect estimates for the constipation–CHD risk association (OR=1.12 vs. 1.10 in primary analysis), suggesting potential underestimation in our original model (Supplementary Figure S1). Moderate heterogeneity was detected (*I*^2^ = 42.5%, *p* = 0.03), with Galbraith plot analysis identifying potential outlier studies contributing to between-study variation (Figure 7A). To evaluate potential study design effects, we performed subgroup analyses stratified by study type. Cohort studies demonstrated a robust association between constipation and elevated CHD risk (HR=1.09, 95% CI: 1.04–1.14), as illustrated in Figure 3A. Geographic subgroup analyses revealed significantly elevated CHD risk associated with constipation across all regions: Americas (HR=1.09, 95% CI: 1.02–1.16), Asia (HR=1.07, 95% CI: 1.02–1.12), and Europe (HR=1.24, 95% CI: 1.14–1.34), as shown in Figure 3B.

**Figure 4:**
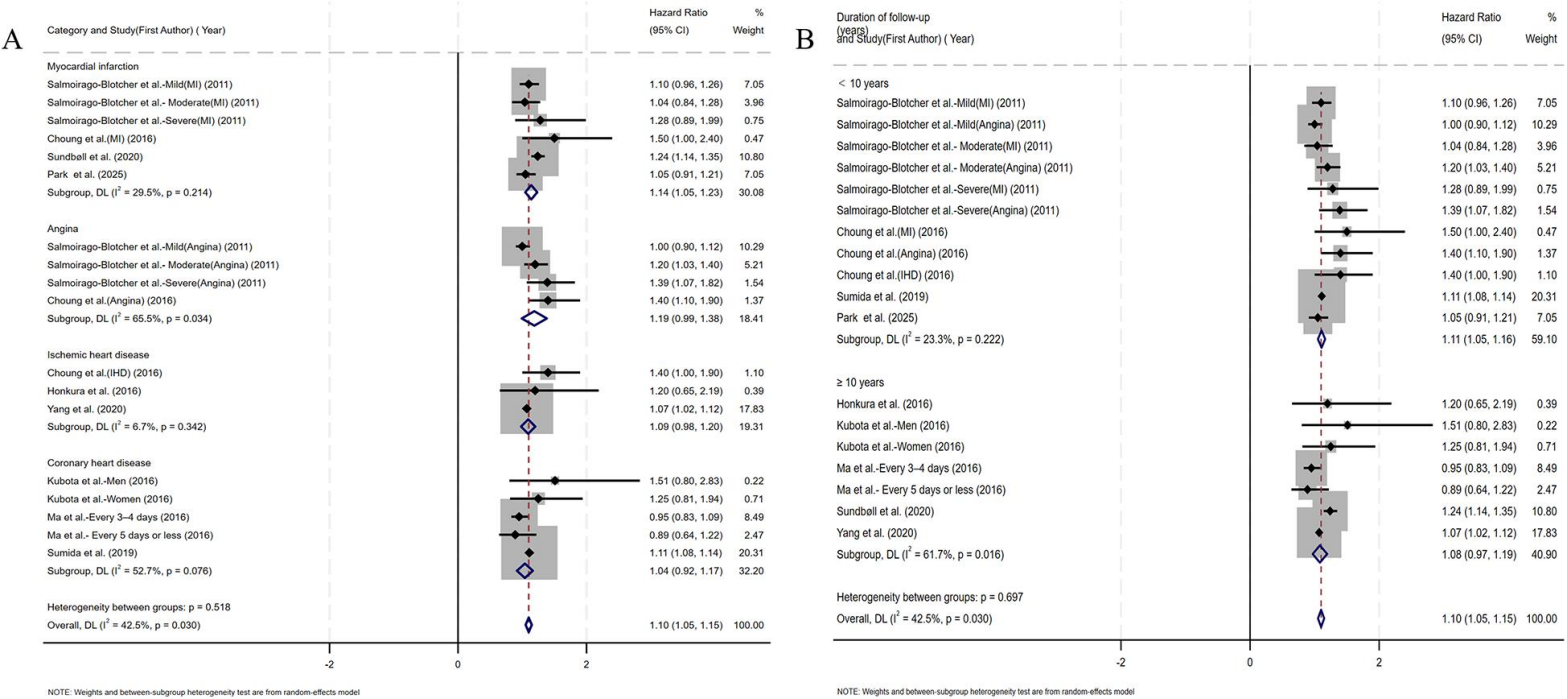
**(A)** Forest plots of subgroup studies regarding the association between constipation and CHD risk based on classification of diseases; **(B)** forest plots of subgroup studies regarding the association between constipation and CHD risk based on follow-up duration.

**Figure 5:**
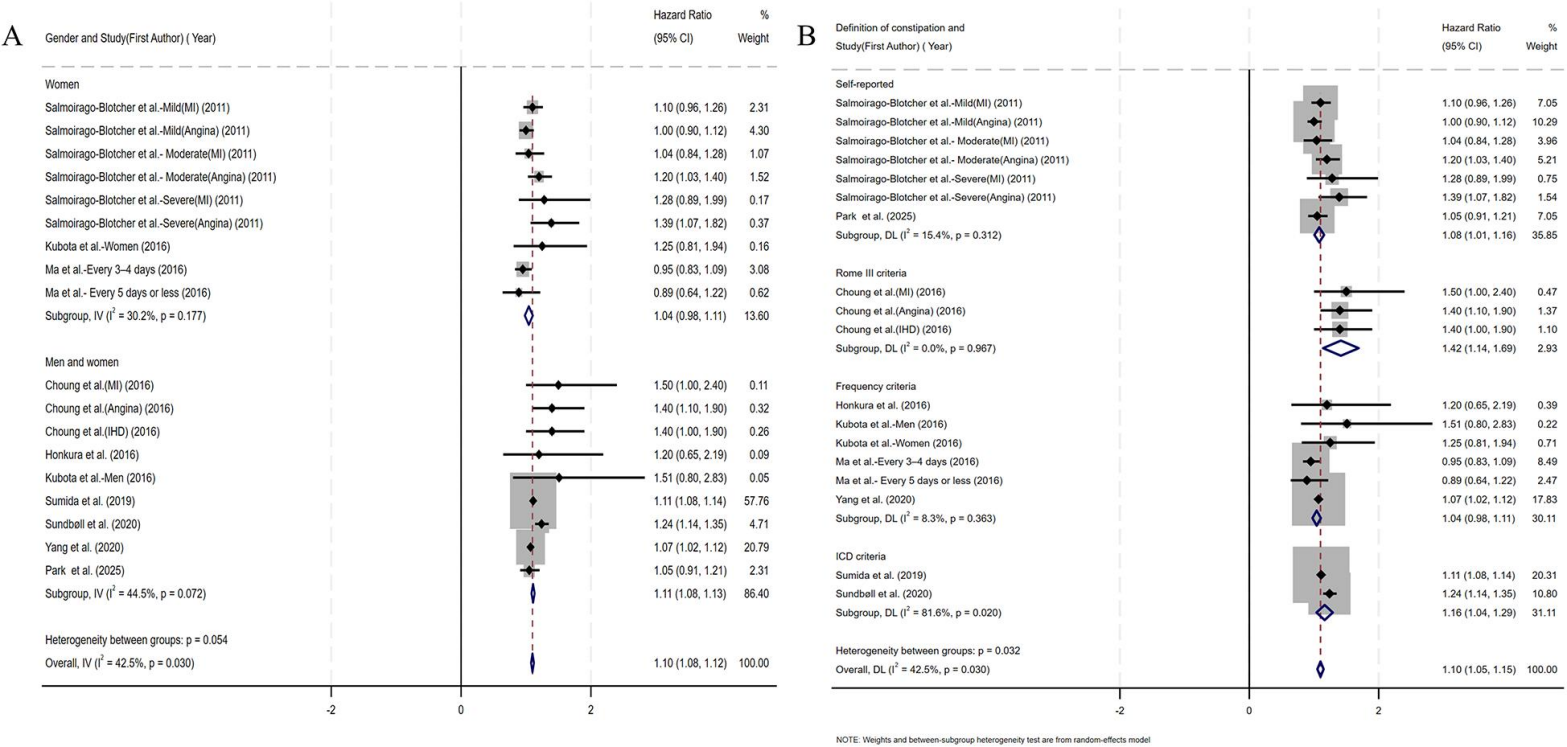
**(A)** Forest plots of subgroup studies regarding the association between constipation and CHD risk based on gender; **(B)** forest plots of subgroup studies regarding the association between constipation and CHD risk based on the definition of constipation.

Coronary artery obstruction represents only one component in the multifactorial pathogenesis of ischemic heart disease, yet most prior studies have not rigorously differentiated ischemic heart disease from CHD terminologically (17). Accordingly, we adopted an operational classification strategy that aligned subgroup definitions with the specific nomenclature used by the original investigators. Subgroup analysis by CHD category demonstrated a significant association between constipation and myocardial infarction risk (HR=1.14, 95% CI: 1.05–1.23). No significant associations were observed for angina pectoris, ischemic heart disease, or coronary heart disease (Figure 4A). Given the substantial reduction in heterogeneity (*I*^2^ < 25%) within the ischemic heart disease subgroup, we performed additional fixed-effect meta-analysis. This analysis confirmed a significant constipation–ischemic heart disease association (HR=1.07, 95% CI: 1.02–1.12), as detailed in Supplementary Figure S2. Stratified by follow-up duration, constipated individuals with <10 years of follow-up demonstrated significantly elevated CHD risk (HR=1.11, 95% CI: 1.05–1.16), whereas no statistically significant association emerged in cohorts with ≥10 years of surveillance (HR=1.08, 95% CI: 0.97–1.19) (Figure 4B). Given the sex-specific design of included studies, we conducted sex-stratified analyses evaluating constipation–CHD associations. The female-specific analysis revealed a non-significant association (HR=1.04, 95% CI: 0.98–1.11) with reduced residual heterogeneity (*I*^2^ = 30.2%, *p* = 0.177), as detailed in Figure 5A. Of the nine cohort studies included in the analysis, only one provided sex-stratified risk estimates specific to male patients, which precluded a meaningful subgroup analysis for this population. Recognizing that heterogeneous definitions of constipation could introduce substantial variability into the pooled estimates, we performed a subgroup analysis stratified by the specific diagnostic criteria used to define constipation across the included studies (Figure 5B). Our results showed that patients diagnosed with constipation according to self-reported constipation (via validated questionnaires or patient interviews), Rome III criteria, and ICD criteria have a significantly increased risk of CHD (HR=1.08, 95% CI: 1.01–1.16; HR=1.42, 95% CI: 1.14–1.69; HR=1.16, 95% CI: 1.04–1.29). In the subgroup diagnosed with constipation according to frequency criteria, although heterogeneity was significantly reduced, constipation was not observed to increase CHD risk (HR=1.04, 95% CI: 0.98–1.11).

### Sensitivity analysis and publication bias

**Figure 6:**
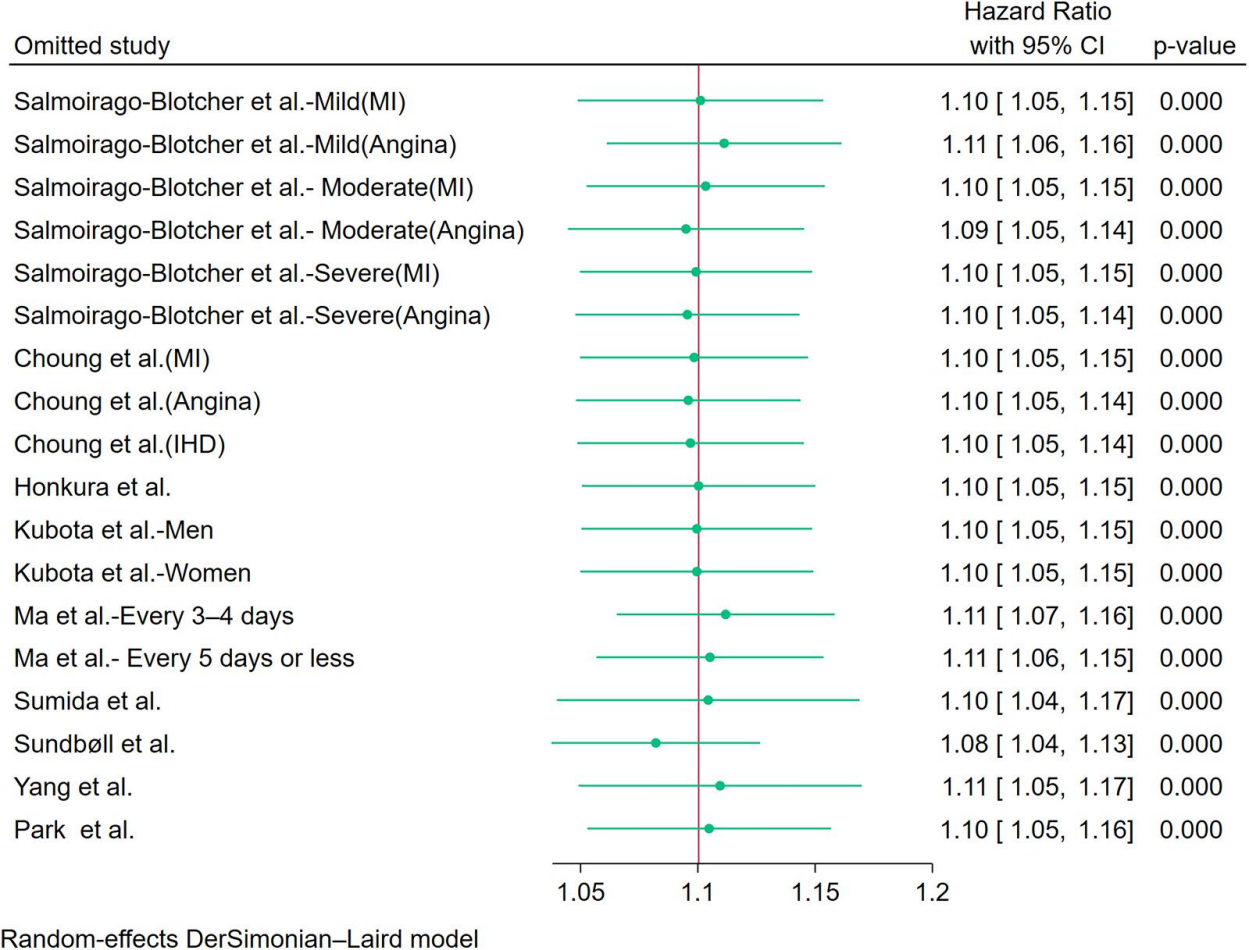
Sensitivity analysis of the included studies.

**Figure 7:**
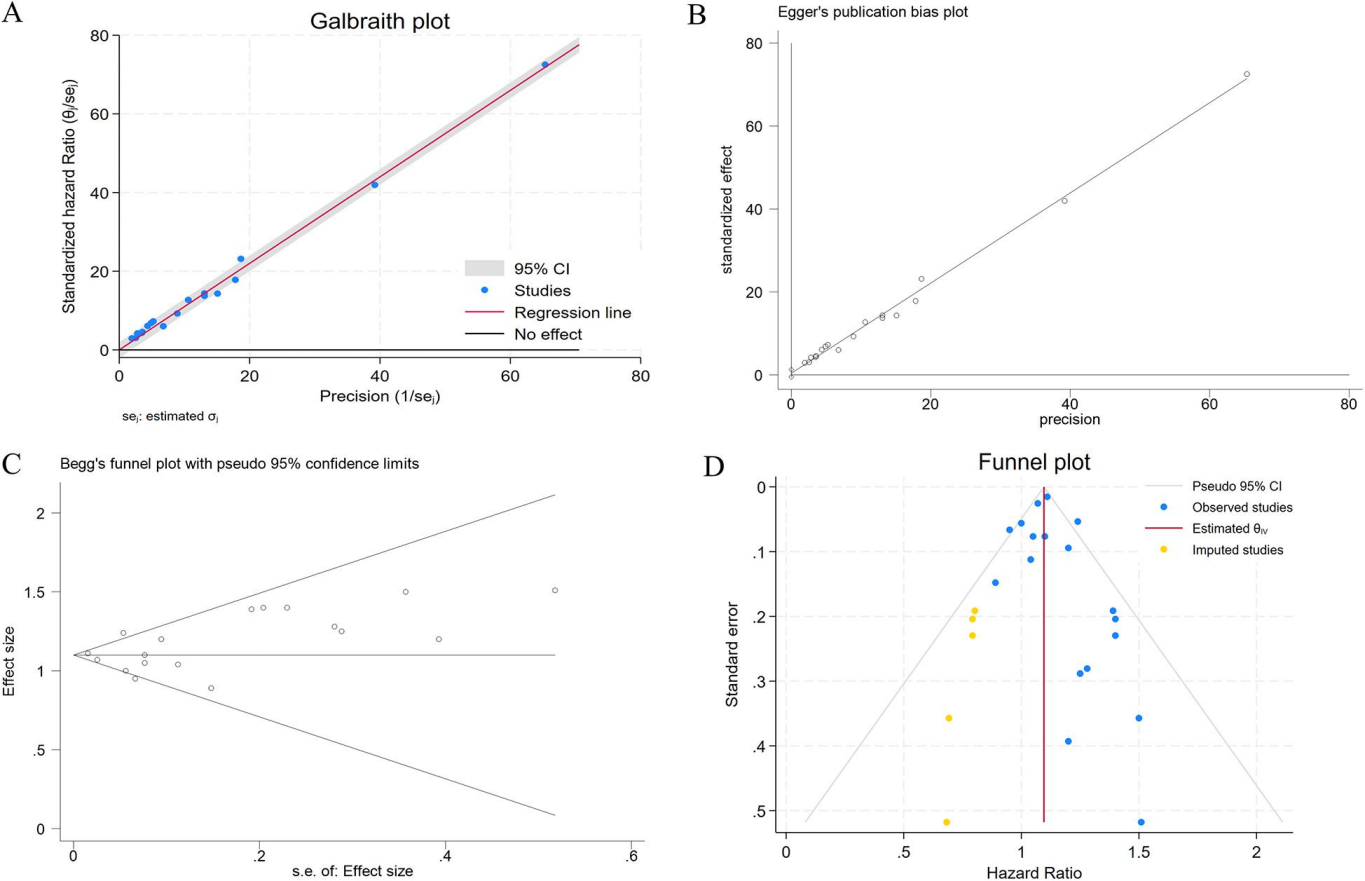
**(A)** The Galbraith plot regarding the primary source of heterogeneity; **(B)** evaluation of publication bias using Egger's test; **(C)** evaluation of publication bias using Begg's funnel plot; **(D)** adjusted funnel plot based on the non-parametric trim-and-fill analysis of publication bias.

Sensitivity analysis using leave-one-out iteration demonstrated robustness of the constipation–CHD association, with all recalculated pooled estimates remaining statistically significant (HR range=1.04–1.17) (Figure 6). This consistency confirms the stability of our meta-analytic findings. To address potential publication bias, we implemented a three-tiered assessment protocol: (1) visual inspection of funnel plot asymmetry, (2) Begg's rank correlation test, and (3) Egger's weighted regression test. Although there was no statistical evidence of publication bias using Egger's test (*p* = 0.340) (Figure 7B) and Begg's test (*p* = 0.343) (Figure 7C), initial funnel plot asymmetry prompted non-parametric trim-and-fill analysis. This method estimates the number of theoretically missing studies required to achieve funnel plot symmetry, incorporating five imputed studies in our analysis (Figure 7D). The adjusted model maintained statistical significance, showing an 8% elevated CHD risk in constipated individuals (HR=1.08, 95% CI: 1.03–1.14).

## Discussion

Our meta-analysis incorporating nine cohort studies demonstrated a consistent risk elevation for CHD among constipation patients (HR=1.10, 95% CI: 1.05–1.15). Geographically stratified analyses revealed comparable effect magnitudes across regions: Americas, Asia, and Europe. Disease-stratified analysis revealed differential associations: myocardial infarction exhibited heightened susceptibility (HR=1.14, 95% CI: 1.05–1.23), whereas angina pectoris showed no statistically significant relationship. Notably, sex-specific effects emerged, with null association observed in female subgroups (HR=1.04, 95% CI: 0.98–1.11).

CHD constitutes the predominant etiology of cardiovascular mortality across both genders, accounting for >50% of total cardiovascular disease burden (18). CHD arises from progressive atherosclerotic transformation of the coronary vasculature, pathologically characterized by endothelial barrier compromise, chronic inflammatory infiltrates, and focal accumulation of lipid-rich macrophage foam cells. Structural instability in high-risk plaques precipitates fibrous cap fissure, initiating platelet-rich thrombus formation and subsequent acute coronary syndrome manifestations (19). The pathophysiology of CHD has undergone a remarkable evolution over the past decade. Formerly characterized as a passive cholesterol accumulation disorder, contemporary models delineate atherogenesis as a complex interaction of risk factors including cells of the artery wall and the blood and molecular messages that they exchange (20). Modern clinical evidence establishes CHD as a preventable disease, where pre-emptive screening coupled with risk-tailored interventions demonstrates significant prognostic benefits, across both established patients and asymptomatic high-risk populations (21). At present, the traditional risk factors of CHD, such as hypertension, diabetes, hyperlipidemia, behavior (tobacco use, physical inactivity), and diet (excessive sodium intake, sugar-sweetened beverage consumption), have been widely concerned (22). Our meta-analysis demonstrated a 10% elevated risk of CHD events among individuals with constipation compared to non-constipated controls, providing epidemiological evidence supporting constipation as a modifiable risk determinant in cardiovascular prevention frameworks. To our knowledge, our study is the first systematic review and meta-analysis to examine the association between constipation and CHD risk.

Constipation represents a prevalent gastrointestinal disorder encountered in clinical settings across Western nations, with epidemiological studies estimating a global prevalence in the range of 12%–19% (23). Constipation is more frequent in North America and Europe compared to Asia, probably attributed to variations in cultural practices, dietary patterns, and environmental exposures (24). Based on colonic transit, it can be categorized into three subtypes: normal colonic transit, rectal evacuation disorders, and slow colonic constipation (25). The multifactorial pathophysiology of constipation complicates precise alignment of causative factors with these subtypes.

The exact mechanism behind the increased risk of CHD observed in patients with constipation in our study is still unclear, and we speculate that it may be mediated through multiple pathways. First, the gut–heart axis, which serves as a pivotal bidirectional communication axis between the gut and the heart, may play an important role in the connection between constipation and CHD (26). Intestinal changes, such as dysbiosis and altered permeability of the epithelial barrier, have been observed in patients with constipation (27). Emerging evidence delineates compromised intestinal barrier integrity, dysregulated microbial communities, and microbiota-derived bioactive metabolites as pivotal mediators contribute to the occurrence and development of CHD via the gut–heart axis pathway (26). The gut microbiota promotes the development of atherosclerosis by producing intermediate metabolites, including trimethylamine-N-oxide (TMAO), endotoxin and phenylacetyl glutamine, and reducing butyrate (28). The most compelling evidence that links gut microbiota and CAD is related to microbial metabolism of dietary factors such as carnitine and choline. The source of TMAO is trimethylamine, which is produced by the gut microbiota from nutrients containing L-carnitine or phosphatidylcholine, and subsequently oxidized to TMAO by hepatic flavin-containing monooxygenases (29–31). Current evidence underscores that TMAO precursors drive foam cell formation and atherosclerotic plaque progression, while TMAO itself induces dysregulation of cholesterol homeostasis, elicits proinflammatory responses, oxidative stress, and endothelial activation—collectively synergizing to advance CHD pathogenesis (26, 30). Second, constipation contributes to sustained hypertension through psychogenic stress, enhanced colonic water absorption, and gut dysbiosis-induced neuroinflammatory signaling (11, 32). During defecation, constipation can often cause straining, during which patients may breathe in a strained manner similar to the Valsalva maneuver (33). This may induce transient blood pressure elevation, potentially precipitating acute coronary syndrome events. Third, constipated patients have upregulated biosynthesis and release of serotonin, a vasoconstrictor that promotes thrombus formation and is related to the development of atherosclerotic plaques and elevated risks of CHD (34–36).

Notably, our meta-analysis revealed no significant association between constipation and CHD risk in female participants, a finding warranting further mechanistic exploration. We hypothesized that pathophysiological mechanisms intrinsic to the predominant constipation phenotype in women underpin this observation. As previously noted, constipation exhibits three distinct phenotypes, with women being more likely to be affected by functional defecatory disorders than men (37). Among women, dyssynergic defecation is the most prevalent subtype and isolated slow transit constipation is uncommon (38). We postulate that accelerated colonic transit in dyssynergic defecation may reduce microbial fermentation time, thereby mitigating gut dysbiosis, compared to slow transit constipation. This diminished dysbiosis could attenuate the constipation–CHD association in women by reducing pathogenic microbial metabolite production (e.g., TMAO) and associated endothelial dysfunction. Subgroup analysis by classification of diseases revealed no significant constipation–CHD risk associations in angina pectoris or CHD subgroups, potentially attributed to sex distribution imbalances (female predominance) within these populations. We also did not observe an increased CHD risk for constipation in the subgroup diagnosed with constipation according to frequency criteria. Unlike definitions based on self-report, Rome III, or ICD codes, which often incorporate symptoms of straining, incomplete evacuation, or hard stools, a frequency-based definition likely identifies a more heterogeneous population. This group may include individuals with transient, diet-related, or behavioral bowel patterns who do not share the underlying autonomic dysregulation, chronic inflammation, or gut microbiome alterations associated with long-term, pathophysiologically significant constipation. Consequently, while frequency-based criteria may reduce heterogeneity by providing an objective measure, they might also dilute the effect by including many individuals without the persistent gut dysfunction that potentially drives CHD risk. In addition, a subgroup with ≥10 years of follow-up showed no significant association between constipation and CHD risk. This null finding may reflect residual confounding from sex-specific factors, laxative exposure duration, and age-related comorbidities.

This meta-analysis has several limitations that require cautious interpretation. First, statistical heterogeneity was observed (*I*^2^ = 42.5%, *p* = 0.03), potentially attributable to be factors such as region, disease classification, follow-up time, gender, diet, and medication use. Subgroup analyses based on gender and region showed reduced heterogeneity, confirming these factors as potential confounders. Second, the limited number of studies in subgroup analyses by disease classification means that further research is needed to validate the association between constipation and different CHD subtypes. Third, due to an insufficient number of studies, a formal dose–response analysis between bowel movement frequency categories and CHD risk could not be performed. Fourth, sex-specific subgroup studies are limited, and we did not analyze constipation and CHD risks in men, highlighting the need for future prospective studies with pre-specified sex-stratified analyses.

## Conclusion

Our meta-analysis demonstrated a significant positive association between constipation and CHD risk (OR=1.10, 95% CI: 1.05–1.15). Further subgroup analysis found that patients with constipation had a 14% higher risk of myocardial infarction compared to non-constipated individuals. These findings suggest that constipation may either accelerate the pathological processes underlying CHD or that both conditions share common etiological pathways. Future studies are warranted to investigate the risk of CHD in patients with constipation and to explore the factors driving this association, in order to confirm and expand upon these results.

## Supplementary material

Supplementary Figure S1 Forest plot of meta-analysis showing the association between constipation and CHD risk (includes three studies reporting only OR for constipation and CHD risk).

Supplementary Figure S2 Forest plots of subgroup analyses showing the association between constipation and CHD risk by disease classification (fixed-effect models).

## References

[1] Roth GA Mensah GA Johnson CO Addolorato G Ammirati E Baddour LM Global burden of cardiovascular diseases and risk factors, 1990–2019: update from the GBD 2019 study. J Am Coll Cardiol. (2020) 76:2982–3021. 10.1016/j.jacc.2020.11.010 33309175 PMC7755038

[2] Vaduganathan M Mensah GA Turco JV Fuster V Roth GA. The global burden of cardiovascular diseases and risk: a compass for future health. J Am Coll Cardiol. (2022) 80:2361–71. 10.1016/j.jacc.2022.11.005 36368511

[3] Park SC Jung J Kwon YE Baeg SI Oh DJ Kim DH Constipation and risk of death and cardiovascular events in patients on hemodialysis. Kidney Res Clin Pract. (2025) 44:155–63. 10.23876/j.krcp.24.174 39815794 PMC11838856

[4] Yang S Yu C Guo Y Bian Z Fan M Yang L Bowel movement frequency and risks of major vascular and non-vascular diseases: a population-based cohort study among Chinese adults. BMJ Open. (2020) 10:e031028. 10.1136/bmjopen-2019-031028 31924633 PMC6955483

[5] Sundbøll J Szépligeti SK Adelborg K Szentkúti P Gregersen H Sørensen HT. Constipation and risk of cardiovascular diseases: a Danish population-based matched cohort study. BMJ Open. (2020) 10:e037080. 10.1136/bmjopen-2020-037080 PMC7473662 32873621

[6] Sumida K Molnar MZ Potukuchi PK Thomas F Lu JL Yamagata K Constipation and risk of death and cardiovascular events. Atherosclerosis. (2019) 281:114–20. 10.1016/j.atherosclerosis.2018.12.021 30658186 PMC6399019

[7] Barberio B Judge C Savarino EV Ford AC. Global prevalence of functional constipation according to the Rome criteria: a systematic review and meta-analysis. Lancet Gastroenterol Hepatol. (2021) 6:638–48. 10.1016/S2468-1253(21)00111-4 34090581

[8] Ford AC Moayyedi P Lacy BE Lembo AJ Saito YA Schiller LR American college of gastroenterology monograph on the management of irritable bowel syndrome and chronic idiopathic constipation. Am J Gastroenterol. (2014) 109(Suppl 1):S2–26; quiz S27. 10.1038/ajg.2014.187 25091148

[9] Ma W Li Y Heianza Y Staller KD Chan AT Rimm EB Associations of bowel movement frequency with risk of cardiovascular disease and mortality among us women. Sci Rep. (2016) 6:33005. 10.1038/srep33005 27596972 PMC5011651

[10] Peng Y Liu F Qiao Y Wang P Ma B Li L Association of abnormal bowel health with major chronic diseases and risk of mortality. Ann Epidemiol. (2022) 75:39–46. 10.1016/j.annepidem.2022.09.002 36116757

[11] Judkins CP Wang Y Jelinic M Bobik A Vinh A Sobey CG Association of constipation with increased risk of hypertension and cardiovascular events in elderly Australian patients. Sci Rep. (2023) 13:10943. 10.1038/s41598-023-38068-y 37414864 PMC10326061

[12] Zheng T Camargo Tavares L D'Amato M Marques FZ. Constipation is associated with an increased risk of major adverse cardiac events in a UK population. Am J Physiol Heart Circ Physiol. (2024) 327:H956–64. 10.1152/ajpheart.00519.2024 39150392

[13] Salmoirago-Blotcher E Crawford S Jackson E Ockene J Ockene I. Constipation and risk of cardiovascular disease among postmenopausal women. Am J Med. (2011) 124:714–23. 10.1016/j.amjmed.2011.03.026 21663887 PMC3144272

[14] Choung RS Rey E Richard Locke G 3rd Schleck CD Baum C Zinsmeister AR Chronic constipation and co-morbidities: a prospective population-based nested case-control study. United Eur Gastroenterol J. (2016) 4:142–51. 10.1177/2050640614558476 PMC4766536 26966534

[15] Honkura K Tomata Y Sugiyama K Kaiho Y Watanabe T Zhang S Defecation frequency and cardiovascular disease mortality in Japan: the Ohsaki cohort study. Atherosclerosis. (2016) 246:251–6. 10.1016/j.atherosclerosis.2016.01.007 26812003

[16] Kubota Y Iso H Tamakoshi A. Bowel movement frequency, laxative use, and mortality from coronary heart disease and stroke among Japanese men and women: the Japan collaborative cohort (JACC) study. J Epidemiol. (2016) 26:242–8. 10.2188/jea.JE20150123 26725286 PMC4848322

[17] Marzilli M Merz CN Boden WE Bonow RO Capozza PG Chilian WM Obstructive coronary atherosclerosis and ischemic heart disease: an elusive link!. J Am Coll Cardiol. (2012) 60:951–6. 10.1016/j.jacc.2012.02.082 22954239

[18] Roger VL Go AS Lloyd-Jones DM Adams RJ Berry JD Brown TM Heart disease and stroke statistics–2011 update: a report from the American Heart Association. Circulation. (2011) 123:e18–e209. 10.1161/CIR.0b013e3182009701 21160056 PMC4418670

[19] Batty JA Subba S Luke P Gigi LW Sinclair H Kunadian V. Intracoronary imaging in the detection of vulnerable plaques. Curr Cardiol Rep. (2016) 18:28. 10.1007/s11886-016-0705-1 26879196 PMC4754333

[20] Libby P Theroux P. Pathophysiology of coronary artery disease. Circulation. (2005) 111:3481–8. 10.1161/CIRCULATIONAHA.105.537878 15983262

[21] Arnett DK Blumenthal RS Albert MA Buroker AB Goldberger ZD Hahn EJ 2019 ACC/AHA guideline on the primary prevention of cardiovascular disease: a report of the American College of Cardiology/American Heart Association task force on clinical practice guidelines. Circulation. (2019) 140:e596–646. 10.1161/CIR.0000000000000678 30879355 PMC7734661

[22] Roth GA Mensah GA Fuster V. The global burden of cardiovascular diseases and risks: a compass for global action. J Am Coll Cardiol. (2020) 76:2980–1. 10.1016/j.jacc.2020.11.021 33309174

[23] Włodarczyk J Waśniewska A Fichna J Dziki A Dziki Ł Włodarczyk M. Current overview on clinical management of chronic constipation. J Clin Med. (2021):10(8):1738. 10.3390/jcm10081738 33923772 PMC8073140

[24] Daniali M Nikfar S Abdollahi M. An overview of interventions for constipation in adults. Expert Rev Gastroenterol Hepatol. (2020) 14:721–32. 10.1080/17474124.2020.1781617 32772745

[25] Black CJ Ford AC. Chronic idiopathic constipation in adults: epidemiology, pathophysiology, diagnosis and clinical management. Med J Aust. (2018) 209:86–91. 10.5694/mja18.00241 29996755

[26] Rivera K Gonzalez L Bravo L Manjarres L Andia ME. The gut-heart axis: molecular perspectives and implications for myocardial infarction. Int J Mol Sci. (2024) 25:12465. 10.3390/ijms252212465 39596530 PMC11595032

[27] Filippone A Ardizzone A Bova V Lanza M Casili G Cuzzocrea S A combination of xyloglucan, pea protein and chia seed ameliorates intestinal barrier integrity and mucosa functionality in a rat model of constipation-predominant irritable bowel syndrome. J Clin Med. (2022) 11(23):7073. 10.3390/jcm11237073 36498647 PMC9739531

[28] Chen W Zhang S Wu J Ye T Wang S Wang P Butyrate-producing bacteria and the gut-heart axis in atherosclerosis. Clin Chim Acta. (2020) 507:236–41. 10.1016/j.cca.2020.04.037 32376324

[29] Tang WH Wang Z Levison BS Koeth RA Britt EB Fu X Intestinal microbial metabolism of phosphatidylcholine and cardiovascular risk. N Engl J Med. (2013) 368:1575–84. 10.1056/NEJMoa1109400 23614584 PMC3701945

[30] Wang Z Klipfell E Bennett BJ Koeth R Levison BS Dugar B Gut flora metabolism of phosphatidylcholine promotes cardiovascular disease. Nature. (2011) 472:57–63. 10.1038/nature09922 21475195 PMC3086762

[31] Koeth RA Wang Z Levison BS Buffa JA Org E Sheehy BT Intestinal microbiota metabolism of l-carnitine, a nutrient in red meat, promotes atherosclerosis. Nat Med. (2013) 19:576–85. 10.1038/nm.3145 23563705 PMC3650111

[32] Merkel IS Locher J Burgio K Towers A Wald A. Physiologic and psychologic characteristics of an elderly population with chronic constipation. Am J Gastroenterol. (1993) 88:1854–9. 8237932

[33] Ishiyama Y Hoshide S Mizuno H Kario K. Constipation-induced pressor effects as triggers for cardiovascular events. J Clin Hypertens. (2019) 21:421–5. 10.1111/jch.13489 PMC8030287 30761728

[34] Costedio MM Coates MD Brooks EM Glass LM Ganguly EK Blaszyk H Mucosal serotonin signaling is altered in chronic constipation but not in opiate-induced constipation. Am J Gastroenterol. (2010) 105:1173–80. 10.1038/ajg.2009.683 20010921 PMC2872481

[35] Hara K Hirowatari Y Yoshika M Komiyama Y Tsuka Y Takahashi H. The ratio of plasma to whole-blood serotonin may be a novel marker of atherosclerotic cardiovascular disease. J Lab Clin Med. (2004) 144:31–7. 10.1016/j.lab.2004.03.014 15252405

[36] Vikenes K Farstad M Nordrehaug JE. Serotonin is associated with coronary artery disease and cardiac events. Circulation. (1999) 100:483–9. 10.1161/01.CIR.100.5.483 10430761

[37] Prichard DO Fetzer J. Recto-anal pressures in constipated men and women undergoing high-resolution anorectal manometry. Dig Dis Sci. (2023) 68:922–30. 10.1007/s10620-022-07590-w 35727425

[38] Ribas Y Saldaña E Martí-Ragué J Clavé P. Prevalence and pathophysiology of functional constipation among women in Catalonia, Spain. Dis Colon Rectum. (2011) 54:1560–9. 10.1097/DCR.0b013e31822cb5c2 22067186

