# Supplementary figures and images for: Association between constipation and risk of coronary heart disease: a systematic review and meta-analysis of cohort studies
Source: Front Cardiovasc Med. 2025 Dec 4;12:1622801. doi: 10.3389/fcvm.2025.1622801 (PMC12711805; doi:10.3389/fcvm.2025.1622801)

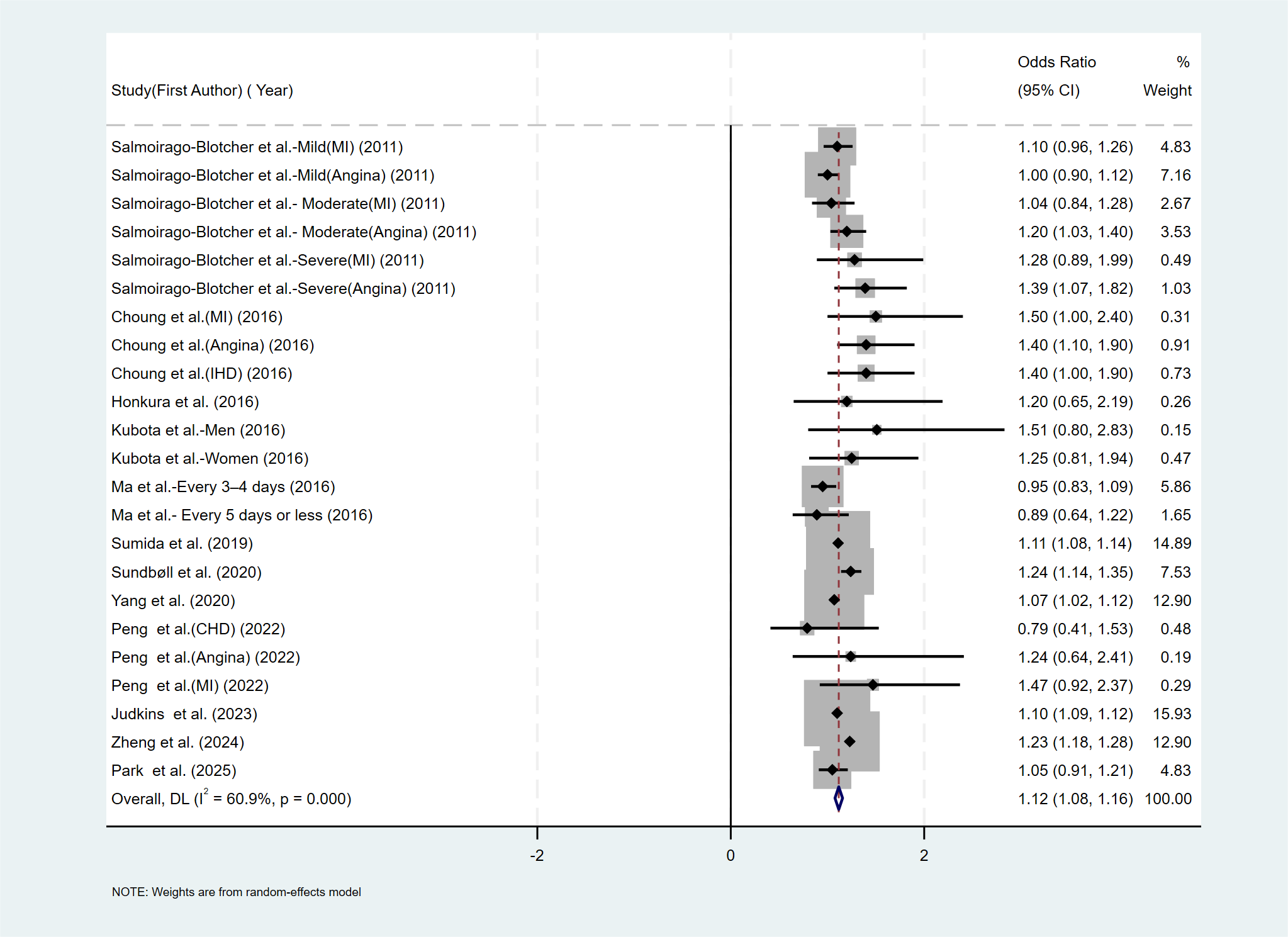

Supplement: Supplementary Figure S1 — Forest plot of meta-analysis showing the association between constipation and CHD risk (includes three studies reporting only OR for constipation and CHD risk). [file Image1.tif]

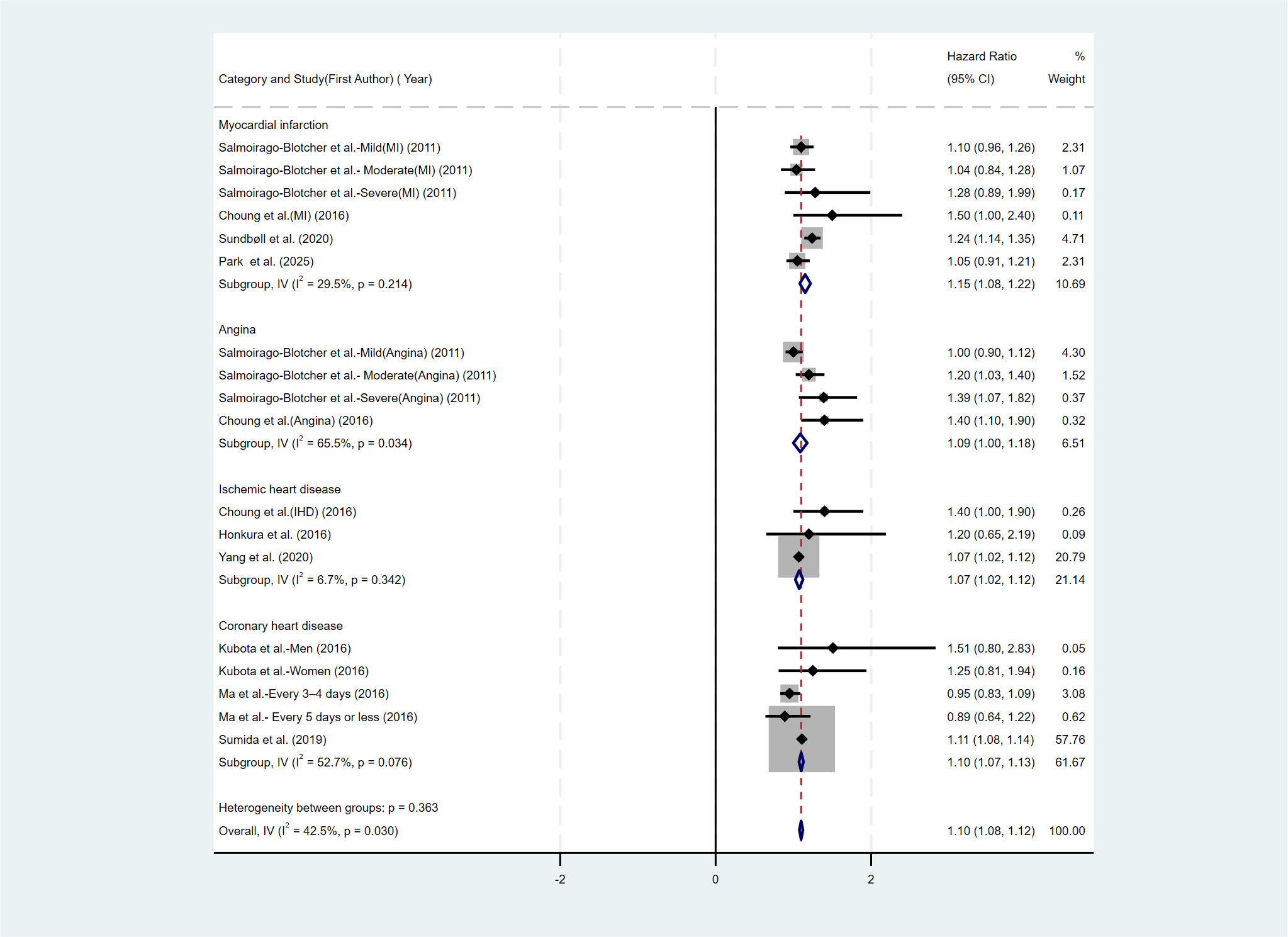

Supplement: Supplementary Figure S2 — Forest plots of subgroup analyses showing the association between constipation and CHD risk by disease classification (fixed-effect models). [file Image2.tif]
