# Supplementary material for: Association between constipation and risk of coronary heart disease: a systematic review and meta-analysis of cohort studies
Source: Front Cardiovasc Med. 2025 Dec 4;12:1622801. doi: 10.3389/fcvm.2025.1622801 (PMC12711805; doi:10.3389/fcvm.2025.1622801)
Supplement: Supplementary file 3 [file Table1.pdf]

Supplementary Material. Concise summary for included studies

Study	Concise summary
Salmoirago-Blotcher et al.	In the prospective cohort study involving 73,047 postmenopausal women from the Women's Health Initiative, constipation was assessed via self-report and examined in relation to cardiovascular disease(CVD) outcomes over a median follow-up of 6.9 years. While unadjusted analyses indicated that moderate and severe constipation were associated with elevated CVD risk, these associations were substantially attenuated after sequential adjustment for demographic, clinical, dietary, and psychosocial variables.
Choung et al.	In the prospective population-based nested case-control study, the association between chronic constipation (defined by Rome III criteria) and various comorbidities was systematically investigated administered to a random community sample. The study demonstrated several methodological strengths relevant to quality assessment, including its population-based design reducing selection bias, age- and gender-matched case-control selection ensuring comparability, and comprehensive medical record abstraction with blinded status adjudication enhancing outcome validity. Key findings showed a modest association between constipation and CVD such as angina pectoris and myocardial infarction.
Honkura et al.	In the large-scale prospective Ohsaki cohort study, which included 45,112 Japanese adults followed for 13.3 years, defecation frequency was assessed via a baseline self-administered questionnaire and its association with CVD mortality was rigorously examined. The study demonstrated several methodological strengths pertinent to quality assessment: it utilized a population-based design with a high participation rate (95%), employed objective mortality data obtained from national health insurance records, and adjusted extensively for a wide range of potential confounders including demographic, lifestyle, and clinical factors. The findings revealed a significant dose-response relationship, whereby less frequent defecation was associated with elevated risks of total CVD mortality and stroke mortality, independent of conventional risk factors.
Kubota et al.	In the large-scale Japan Collaborative Cohort (JACC) Study, which prospectively followed 72,014 Japanese participants over a median of 20 years, the associations between bowel movement frequency, laxative use, and CVD mortality were examined using Cox proportional hazards models. The study demonstrated several methodological strengths relevant to bias control: it employed a population-based design with systematic enrollment across 45 regions, utilized validated mortality surveillance through national death certificates, and comprehensively adjusted for a wide range of potential confounders including demographic, lifestyle, and clinical factors. The findings indicated that significant association between bowel movement frequency and mortality from CVD was not observed.
Ma et al.	In the prospective cohort analysis using data from the Nurses' Health Study, the relationship between bowel movement frequency and CVD risk was evaluated among 86,289 US women over 30 years of follow-up. The study demonstrated several methodological strengths pertinent to bias minimization: it utilized a well-defined cohort of healthcare professionals, achieved high follow-up rates (>90%), employed validated questionnaires for exposure assessment, and ascertained outcomes via medical record review with physician adjudication to reduce misclassification. The findings revealed that more frequent bowel movements (>1/day) were associated with higher risks of total mortality after adjustment for BMI and diabetes, though associations with incident CVD were attenuated upon full adjustment.

Sumida et al.	In the large-scale retrospective cohort study involving 3,359,653 U.S. veterans, the association between constipation and cardiovascular outcomes was rigorously examined over a median follow-up of 6.7 years. It utilized a well-defined, nationally representative cohort from the VA healthcare system, ensuring minimal selection bias through comprehensive inclusion of patients with baseline eGFR ⩾60 mL/min/1.73 m^2. To enhance comparability, the authors employed multivariable adjustment for an extensive set of confounders, including demographics, comorbidities, medications, and socioeconomic factors, and conducted propensity score matching as a sensitivity analysis.The results indicated that constipation was independently associated with increased risks of all-cause mortality, incident coronary heart disease, and ischemic stroke.
Sundbøll et al.	In the Danish population-based matched cohort study, the study leveraged nationwide registry data encompassing the entire Danish population; cases were identified using hospital-based diagnoses of constipation from the Danish National Patient Registry, while controls were meticulously matched from the general population on age, sex, and calendar year. For comparability, the design inherently addressed key confounders through matching, and the analysis further adjusted for an extensive array of potential confounders—including comorbidities, medications, and socioeconomic factors—via multivariable Cox models, thereby robustly controlling for confounding. In terms of outcome assessment, follow-up was virtually complete due to Denmark's comprehensive civil registration system, eliminating loss-to-follow-up bias and ensuring accurate outcome detection. The results indicated that constipation was associated with an increased risk of several cardiovascular diseases.
Yang et al.	In the large-scale prospective China Kadoorie Biobank study, which included 487,198 Chinese adults followed for a median of 10 years, bowel movement frequency (BMF) was assessed via baseline self-report and its associations with multiple vascular and non-vascular diseases were examined. Regarding selection bias, the cohort was population-based, drawn from 10 geographically diverse regions in China, ensuring broad representativeness. For comparability, the analysis utilized stratified Cox models with age as the time scale and adjusted for an extensive array of potential confounders, including sociodemographics, lifestyle factors, medical history, and anthropometric measures, effectively addressing key sources of confounding. Concerning outcome assessment, endpoints were ascertained through rigorous linkages with national registries and health insurance databases, with validation of diagnoses using standardized ICD-10 coding by trained personnel, minimizing outcome misclassification. The findings revealed BMF was associated with future risk of multiple vascular and non- vascular diseases,including CVD, COPD, type 2 diabetes and chronic kidney disease.
Park et al.	The nationwide cohort study investigaties the association between constipation and cardiovascular outcomes in 35,230 Korean hemodialysis patients.Regarding selection bias, the study utilized a representative national cohort from the Korean Health Insurance Review and Assessment Service database, encompassing nearly all patients undergoing maintenance hemodialysis in South Korea. Constipation was defined objectively using prescription records. For comparability, the analysis employed multivariate Cox models adjusting for potential confounders, including demographics, dialysis vintage, comorbidities, laboratory parameters, and medication use. In terms of outcome assessment, endpoints were ascertained using validated ICD-10 codes supplemented by procedure codes. The median follow-up of 5.4 years and comprehensive linkage with national registries ensured complete endpoint capture. Results indicated that constipation was associated with increased risks of the primary composite outcome, all-cause mortality and ischemic stroke, though associations with hemorrhagic stroke and myocardial infarction were non-significant after adjustment.
